# Editorial: Motivation for physical activity, volume II

**DOI:** 10.3389/fpsyg.2024.1533943

**Published:** 2024-12-16

**Authors:** Pedro Morouço, Aleksandra M. Rogowska, Behzad Behzadnia

**Affiliations:** ^1^Polytechnic University of Leiria, Leiria, Portugal; ^2^Research Center in Sports Sciences, Health Sciences and Human Development (CIDESD), Leiria, Portugal; ^3^Institute of Psychology, University of Opole, Opole, Poland; ^4^Department of Motor Behaviour, Faculty of Physical Education and Sport Science, University of Tabriz, Tabriz, Iran

**Keywords:** physical activity motivation, self-efficacy, behavioral determinants, intervention strategies, psychological factors, environmental influences, mental health and physical activity

## Introduction

Motivation is a cornerstone of human behavior, directing the choices and actions shaping physical and mental wellbeing. Within the realm of physical activity, understanding the factors that drive participation, and adherence holds profound implications for public health, education, and clinical interventions. Building on the success of the first volume (Rogowska and Morouço, 2024), this Research Topic presents a diverse collection of 10 articles that delve into the psychological, social, and behavioral determinants of physical activity across various populations and contexts. Guided by contemporary theories (e.g., self-determination theory), these studies illuminate the intricate interplay between motivation and physical activity, offering valuable insights for researchers, practitioners, and policymakers.

The contributions in this Research Topic span a broad spectrum of research areas, from theoretical explorations of motivational constructs to practical interventions aimed at fostering physical activity. Collectively, they enrich our understanding of how motivational processes operate in diverse settings and populations.

## Motivation and self-efficacy across life stages

The relationship between self-efficacy and motivation is foundational in understanding physical activity behaviors across different stages of life. Tao et al. investigate the links between self-efficacy and motivation levels among emerging adults through the lens of self-determination theory. Their findings underscore the importance of fostering intrinsic motivation to enhance physical activity levels in this critical developmental stage. Similarly, Grasaas and Sandbakk analyze trends among Norwegian adolescents, revealing positive associations between self-efficacy and adherence to physical activity recommendations.

## Personality and psychological factors

Individual differences in personality and psychological characteristics shape how people engage with physical activity. Zhang W. et al. delve into the dualistic model of passion to examine how self-oriented perfectionism influences exercise participation. This study highlights the nuanced role of personality traits in shaping physical activity behaviors. Complementing this, Zhang Y. et al. explore procrastination's impact on physical activity among university students, identifying the chain-mediated roles of time management and exercise motivation.

## Family and environmental influences

The role of the social and physical environment is crucial in shaping early and sustained engagement in physical activity. The family environment emerges as a critical determinant in Huang et al.'s study, which explores associations between family factors and physical activity clustering in preschool children. This emphasis on early life determinants aligns with the national-level analysis by Guo et al., who examine recreational screen time and its impact on Chinese children and adolescents' activity levels.

## Interventions to enhance physical activity

Intervention strategies remain central to efforts to promote physical activity and improve health outcomes. Innovative interventions are also represented. Gómez-Cuesta et al. evaluate the efficacy of a mobile app-based program in improving fitness and body composition, highlighting its adaptability across varying activity levels. Martinez Kercher et al. investigate the psychological and motivational underpinnings of resistance training outcomes in adults, demonstrating the pivotal role of psychological needs and self-efficacy.

## Educational and clinical contexts

Physical education and mental health contexts offer unique opportunities to study motivation's role in promoting physical activity. The mediating role of motivation is explored in Miao et al.'s study, which highlights how perceived variety-support in physical education enhances learning engagement among middle school students. Meanwhile, Chen et al. provide evidence of the protective effects of physical activity against depression in adolescents, offering critical insights into mental health interventions.

## Broader context and future directions

The insights offered by this Research Topic place the study of physical activity motivation within a broader context of contemporary behavioral science. They reflect the multidimensionality of motivation, emphasizing that sustaining engagement in physical activity demands an integrative approach—one that accounts for individual, social, and environmental factors.

Looking ahead, the field stands poised for significant developments. Emerging technologies, such as AI-driven or web-based personalized interventions, and the growing recognition of cultural and contextual factors, offer promising avenues for further exploration. Additionally, experimental and longitudinal research are crucial to explore the long-term effects of motivational strategies for physical activity adherence. The studies featured in this Research Topic collectively advance our understanding of the complex dynamics that motivate individuals to engage in physical activity. By addressing diverse populations and using various theoretical frameworks, they provide a robust foundation for future research and practice. It is our hope that these contributions will encourage further innovation and collaboration toward healthier, more active societies.
